# ALONE WITH MY THOUGHTS: USING NATURAL LANGUAGE PROCESSING TO DISTINGUISH LONELY FROM CALM ALONENESS

**DOI:** 10.1093/geroni/igad104.1060

**Published:** 2023-12-21

**Authors:** Jennifer Lay

**Affiliations:** University of Exeter, Exeter, England, United Kingdom

## Abstract

Time spent alone is common in daily life, and particularly in old age; it may at times be experienced as lonely, and at times as calming (positive solitude). The use of machine learning to interpret human language (e.g., natural language processing or NLP) has been growing in psychology, and NLP has been used to distinguish experiences of loneliness from positive solitude in social media data (e.g., tweets). How effective might such NLP techniques be for classifying naturally-occurring aloneness experiences in older adults’ everyday lives? The present study uses 1,546 thought samples collected from 133 adults aged 18-85 (M = 49.6 years; 73% female) at moments when they were alone over a 1-3 week period; participants reported their current thoughts (open-ended) and affective states (lonely, calm) at each assessment. Support vector machines were then used to classify moments of aloneness as being lonely (vs. not lonely) and calm (vs. not calm) based on the words participants used when describing their thoughts. When compared against participants’ actual reported affective states, these classifications achieved up to 67% accuracy for “lonely” and 79% accuracy for “calm”. “Lonely” thought reports included the word “work” more frequently, whereas “calm” thought reports more frequently included the words “reading” and “think/thought”, suggesting individuals engage in quiet or contemplative activities during positive solitude. Age differences therein are also examined. Findings suggest that NLP may be a useful tool for identifying older adults at risk of loneliness (and its negative health implications) by using brief reports of their everyday thoughts.

